# Targeting STAT6-mediated synovial macrophage activation improves pain in experimental knee osteoarthritis

**DOI:** 10.1186/s13075-024-03309-6

**Published:** 2024-03-20

**Authors:** Garth Blackler, Yue Lai-Zhao, Joseph Klapak, Holly T. Philpott, Kyle K. Pitchers, Andrew R. Maher, Benoit Fiset, Logan A. Walsh, Elizabeth R. Gillies, C. Thomas Appleton

**Affiliations:** 1 Department of Physiology and Pharmacology, Western University, London, ON N6A 5B5 Canada; 2 Bone and Joint Institute, Western University, London, ON N6A 5B5 Canada; 3 Rosalind and Morris Goodman Cancer Research Centre, McGill University, Montreal, QC H3A 1A3 Canada; 4 Department of Human Genetics, McGill University, Montreal, QC H3A 0C7 Canada; 5 Department of Chemistry, Western University, London, ON N6A 5B5 Canada; 6 Department of Chemical and Biochemical Engineering, Western University, London, ON N6A 5B5 Canada; 7 Department of Medicine, Schulich School of Medicine and Dentistry, Western University, London, ON N6A 5C1 Canada

**Keywords:** Signal transducer and activator of transcription 6, macrophages, pain, osteoarthritis

## Abstract

**Background:**

Pain from osteoarthritis (OA) is one of the top causes of disability worldwide, but effective treatment is lacking. Nociceptive factors are released by activated synovial macrophages in OA, but depletion of synovial macrophages paradoxically worsens inflammation and tissue damage in previous studies. Rather than depleting macrophages, we hypothesized that inhibiting macrophage activation may improve pain without increasing tissue damage. We aimed to identify key mechanisms mediating synovial macrophage activation and test the role of STAT signaling in macrophages on pain outcomes in experimental knee OA.

**Methods:**

We induced experimental knee OA in rats via knee destabilization surgery, and performed RNA sequencing analysis on sorted synovial tissue macrophages to identify macrophage activation mechanisms. Liposomes laden with STAT1 or STAT6 inhibitors, vehicle (control), or clodronate (depletion control) were delivered selectively to synovial macrophages via serial intra-articular injections up to 12 weeks after OA induction. Treatment effects on knee and hindpaw mechanical pain sensitivity were measured during OA development, along with synovitis, cartilage damage, and synovial macrophage infiltration using histopathology and immunofluorescence. Lastly, crosstalk between drug-treated synovial tissue and articular chondrocytes was assessed in co-culture.

**Results:**

The majority of pathways identified by transcriptomic analyses in OA synovial macrophages involve STAT signaling. As expected, macrophage depletion reduced pain, but increased synovial tissue fibrosis and vascularization. In contrast, STAT6 inhibition in macrophages led to marked, sustained improvements in mechanical pain sensitivity and synovial inflammation without worsening synovial or cartilage pathology. During co-culture, STAT6 inhibitor-treated synovial tissue had minimal effects on healthy chondrocyte gene expression, whereas STAT1 inhibitor-treated synovium induced changes in numerous cartilage turnover-related genes.

**Conclusion:**

These results suggest that STAT signaling is a major mediator of synovial macrophage activation in experimental knee OA. STAT6 may be a key mechanism mediating the release of nociceptive factors from macrophages and the development of mechanical pain sensitivity. Whereas therapeutic depletion of macrophages paradoxically increases inflammation and fibrosis, blocking STAT6-mediated synovial macrophage activation may be a novel strategy for OA-pain management without accelerating tissue damage.

**Supplementary Information:**

The online version contains supplementary material available at 10.1186/s13075-024-03309-6.

## Background

Pain from osteoarthritis (OA) is one of the top causes of disability worldwide, but effective treatments are lacking and limited by adverse effects. For e.g., an anti-nerve growth factor (NGF) monoclonal antibody (tanezumab) effectively modified pain, but also increased rates of rapidly progressive OA [1]. Effective targets to modify OA-related pain without accelerating joint damage are urgently needed. Synovial inflammation (synovitis) is dominated by macrophage infiltration and strongly associated with worse pain and joint damage in knee OA patients [2–5]. We and others suspect that synovial macrophages mediate pain outcomes, but the mechanisms controlling synovial macrophage activation in OA are not well understood.

Macrophages reside in healthy synovium and maintain tissue homeostasis through phagocytosis of extracellular matrix turnover products and efferocytosis of dead and dying cells [6, 7]. During OA, peripheral macrophages are recruited to synovial tissue and activated [8], contributing to the genesis of chronic OA-related pain through the release of nociceptive molecules including cytokines that act on sensory nerve fibres and via crosstalk with other cell types [9]. Pro- (M1) or anti-inflammatory (M2) macrophage polarization can be stimulated *in vitro* [10], but exposure to disease-specific cues in the local joint microenvironment leads to a wider range of activation states [11]. This complexity underlies the helpful and harmful roles played by macrophages during chronic inflammation and its resolution.

Synovial macrophages may be a key target for the treatment of OA-related pain [6], but are also required for the maintenance of joint homeostasis. Supporting this catch-22 hypothesis, previous studies have shown that depletion of synovial macrophages (for e.g., with liposomal clodronate or genetic deletion) in animal models of OA leads to a paradoxical increase in inflammation and destruction of joint tissues [12, 13]. Therefore, we hypothesize that inhibiting macrophage activation (instead of ablation) may be an effective strategy to improve pain outcomes while preserving the homeostatic functions of synovial macrophages during OA.

The mechanisms leading to synovial macrophage activation during OA development are not well understood. Interestingly, the signal transducer and activator of transcription (STAT) family plays key roles in regulating macrophage activation, polarization, and crosstalk in other diseases [11, 14]. This suggests that STAT signaling may also be important for macrophage activation in OA, but the role of STAT signaling in OA macrophages in pain and synovial inflammation has not been described. Small molecule inhibitors of intra-cellular signaling mechanisms are routinely used to manage rheumatoid and psoriatic arthritis in the clinic [15]. To test the role of STAT signaling in this study, we packaged highly selective small molecule STAT1 or STAT6 inhibitors in large multi-lamellar liposomes. After intra-articular injection, liposomes and their drug payload are phagocytosed by synovial macrophages, allowing us to selectively target macrophage activation. Our objectives were to identify major mechanisms of synovial macrophage activation and test the role of STAT signaling in macrophages on pain outcomes in experimental knee OA.

## Methods

### Rat model of experimental knee OA

Sprague Dawley rats (Charles River Laboratory, Quebec, Canada, strain code 400) were housed and handled in the Animal Care and Veterinary Services conventional housing facility at Western University in accordance with the guidelines of the Canadian Council on Animal Care. The animal use protocol was approved by the Western University Animal Care and Use Committee (AUP2017-042). Twelve-week-old male rats underwent anterior cruciate ligament transection and destabilization of the medial meniscus surgery (OA), or a sham surgery (control) as previously described [16].

### Synovial macrophage sorting and RNA isolation

Synovial tissue was dissected from the entire knee as previously described [16], pooled from 3 or 4 animals per replicate, providing n=5 replicates per condition at 4 and 12 weeks after joint surgery. After rinsing in PBS, synovial cells were enzymatically dissociated as previously described [7]. CD11b+ cells (monocyte/macrophage) were sorted using magnetic-activated cell sorting separation columns (Miltenyi Biotec) and lysed with TRIzol (Fisher Scientific) for RNA isolation using the Direct-zol RNA MicroPrep Kit (Zymo Research). RNA was analysed using a Qubit 1.0 fluorimeter and the Qubit RNA high sensitivity kit (Fisher Scientific); RNA with integrity numbers 7.8-9.3 were sequenced.

### RNA-sequencing data analysis

RNA sequencing libraries were prepared with the Illumina stranded total RNA prep with ribo-zero plus kit. LabChip (Perkin Elmer) was used for quality assessment and qPCR for quantitation of libraries. Libraries (100 base-pair, paired-end) were sequenced using a NovaSeq 6000 S4 reagent kit v1.5 (Illumina) at the McGill Genome Centre. Fastp (v0.20.0) was used to collect QC metrics of the raw reads [17]. RNA sequences were aligned and sorted by coordinates, to the NCBI rat genome Rattus_Rnor6_V102, using STAR aligner (STAR-2.7.6a) [18]. Alignment duplicates were removed with Sambamba (v0.8.0) [19]. Gene quantification was performed using featureCounts (v2.0.0) [20]. DESeq2 (v1.24.0) was used to normalize feature counts and identify differentially expressed genes [21]. The HGNC symbols were extracted and added to the DESeq2 results using biomaRt (v2.40.4) using the rat "rnorvegicus_gene_ensemb" BioMart version "Ensembl Release 102 (November 2020)" [22, 23]. Differential gene expression for OA versus sham included a log_2_-fold change ≥0.5 and an adjusted p value <0.05. Volcano plots were created using VolcaNoseR and bubbleplots in Rstudio (4.2.0) using ggplot2 [24, 25]. Gene Set Enrichment Analysis (GSEA v4.1.0) was used to identify significantly enriched pathways between OA and sham at each timepoint using the Gene Ontology and Hallmark collections from the Molecular Signatures Database [26]. Enriched pathways with a false discovery rate of <0.05 were considered significant. Venny 2.1.0 was used to compare transcription factor involvement in enriched Hallmark pathways [27].

### Drug-loaded liposomes

Commercial liposomes contained phosphate-buffered saline (Veh-lip; control) or 18.4 mM clodronate (Clod-lip) (Encapsula NanoScience). Liposomes prepared in-house containing fludarabine (STAT1i-lip) (Sigma-Aldrich) or AS1517499 (STAT6i-lip) (Sigma-Aldrich) were spontaneously formed via vesicle dispersion containing a 7:3 molar ratio of L-α-phosphatidylcholine (Avanti Polar Lipids) and cholesterol (Sigma-Aldrich) in chloroform to match the commercial liposomes. The dispersion was rotary evaporated under vacuum and heat. The phospholipid film was hydrated with a 2.5 mg/mL solution of fludarabine (STAT1i-lip) in PBS, whereas AS1517499 (STAT6i-lip) at 1 mg/mL was added prior to phospholipid film preparation due to its hydrophobic nature. Liposomes were extruded 6 times through 1.0 μm pore polypropylene filters to form a liposome suspension with an average diameter of 1.25 μm (0.7-2 μm) matching the commercial liposomes. Liposomes were purified using a 30,000 Dalton centrifugal size-exclusion column (Sigma-Aldrich). To validate shape and structure, STAT1i-lip and STAT6i-lip were fixed in 1.5% paraformaldehyde and 1.5% glutaraldehyde in PBS and imaged on a Philips Electronics CM10 Transmission Microscope with a Hamamatsu ORCA 2MPx HRL Camera (Supplemental Fig. 1A). Dynamic light scattering (Malvern Panalytical, Zetasizer Nano ZS) was used to confirm size distribution in all batches. (Supplemental Fig. 1B).

### Liposome delivery

Liposomes were delivered via intraarticular injection. Rats were anaesthetized, the skin around the joint was prepared for injection using a sterile Hamilton syringe equipped with a 30-gauge needle to deliver 50 μL of liposomes. The needle was oriented to the central line of the joint and inserted 2-3 mm through the infra-patellar fat pad to reach the inter-condylar joint space. Injections were initiated 14 days after surgery, providing a total of 2 injections (day 14 and 21) in the 4-week endpoint group, and 6 injections (day 14, 21, 28, 42, 56, and 70) in the 12-week endpoint group.

### Pressure Application Measurement (PAM)

Mechanical sensitivity at the knee was measured longitudinally in all animals in the 12-week endpoint group (n=10 per condition) as previously described [28]. Briefly, a pressure application measurement (PAM) algometer (Bioseb) was placed on the medial joint line of the knee and pressure was applied at approximately 200 grams per second until a withdrawal or vocalization occurred. Measurements were taken at baseline, 4-, 8-, and 12-week timepoints.

### Electronic von Frey (eVF)

Using the same measurement approach as above, ipsilateral hindpaw mechanosensitivity was measured longitudinally in all 12-week endpoint rats as previously described [28]. Briefly, force was applied to the plantar surface of the hindpaw by the applicator tip of an electronic von Frey instrument (Bioseb). Force was applied at 25 grams per second until withdrawal occurred. Three measurements were taken with a 5-minute recovery period.

### Cartilage and synovial histopathology

Whole knees were harvested for histopathology analysis at 4- and 12-week endpoints from half of the rats included in the liposome treatment cohort (n=5 per condition), fixed, processed, sectioned, and stained with either toluidine blue or haematoxylin & eosin (H&E) as previously described [28]. Toluidine blue-stained sections (3 per animal, collected every ~600 μm to span the mid-tibiofemoral joint) were used for grading using the OARSI Histopathology cartilage degeneration system [29]. Mean grades for each of the medial and lateral femoral condyles and tibial plateaus were summed for a total joint cartilage degeneration score. H&E-stained sections (3 per animal, collected every ~600 μm to span the mid-tibiofemoral joint) were used for grading synovial histopathology using a six-component scoring system [30]. Mean grades for each of the medial and lateral parapatellar and tibiofemoral regions were collected for synovial lining thickness, synovial infiltration, fibrin deposition, vascularization, fibrosis, and perivascular edema. Mean grades from each anatomical region were summed for a total joint score for each component.

### Joint tissue co-culture system

The remaining liposome treated knees were allocated to tissue co-culture testing (n=5 per condition). Whole knee synovial tissue was dissected under sterile conditions and transferred to co-culture as previously described [16]. Briefly, synovial tissue explants were cultured in seated 12-well trans-well inserts (Fisher Scientific) for 24-hours before being transferred together with the associated conditioned media to co-culture with passage 1 naïve rat chondrocytes. After 24 hours, conditioned media was collected to assess sulfated glycosaminoglycan production, synovial tissue was fixed for immunofluorescent analysis, and chondrocytes were lysed with TRIzol for RNA purification and gene expression analysis.

### Sulfated glycosaminoglycan assay

Sulfated glycosaminoglycan (sGAG) content in conditioned medium was assessed as previously described [16]. Briefly, Conditioned medium or controls were diluted 1:2 with dimethyl-methylene blue (Sigma Aldrich) and absorbance at 595nm was measured.

### Chondrocyte RNA isolation and gene expression analysis

Chondrocyte RNA purification and gene expression analysis was performed as previously described [16]. RNA was isolated using the RNAEasy Mini Kit (Qiagen) and phase separation tubes (Fisher Scientific), and reverse transcribed using the iScript Reverse Transcription Supermix (Bio-Rad). Quantitative real-time PCR was performed using SsoAdvanced Universal SYBR Green Supermix (Bio-Rad) with predesigned primers for *Acan, Col2A1, Prg4, Sox9, Adamts5, Mmp3, Mmp13, Ccl2, Il6, and S100A8* (Bio-Rad). Cq values were calculated on CFXMaestro 1.1 (Bio-Rad) and normalized to reference genes prior to 2^(-ΔΔCq)^ analysis.

### Immunofluorescence

Synovial lining macrophages were quantified in liposome-treated joint tissue sections (n = 3 per condition) using immunofluorescence labelling as previously described [7]. Sections were labeled with rabbit anti-CD68 (Abcam) or rabbit polyclonal isotype control (Abcam, ab37415) at 0.005 mg/mL in blocking buffer overnight at 4^o^C. Goat anti-rabbit-Alexa-488 secondary antibody (Jackson ImmunoResearch) at 0.0015 mg/mL was applied before washing and mounting with ProLong Gold Antifade Mountant with DAPI (Fisher Scientific). Slides were imaged on a Zeiss LSM 800 AiryScan confocal microscope using the 40X water immersion lens with configuration settings held constant across all samples. Two regions of interest were imaged per section.

### Statistical Analysis

Mechanical sensitivity measures were analyzed using linear mixed effects regression models for each treatment group (Veh-lip, Clod-lip, STAT1i-lip, STAT6i-lip) as previously described [28]. Using separate models for each behavior tested, time was entered as a categorical predictor variable for fixed effects and animal ID was entered as random intercepts. Assumptions for linear mixed models were tested and likelihood ratio tests and Bayesian Information Criterion were used to evaluate model fit. Measures of association are reported as unstandardized beta (β) coefficients ± 95% confidence intervals (CIs) and standard error. In addition, post estimation pairwise comparisons of treatment to control (Veh-lip) were completed with Sidak correction. Cartilage and synovial histopathology, synovial macrophage density, and chondrocyte co-culture measures of sGAG and gene expression were analyzed with one-way ANOVA in GraphPad Prism (version 9.1.2) with Dunnett multiplicity adjustment. A p-value of ≤0.05 was considered significant.

## Results

### Differential gene expression and gene set enrichment analysis

**Fig. 1:**
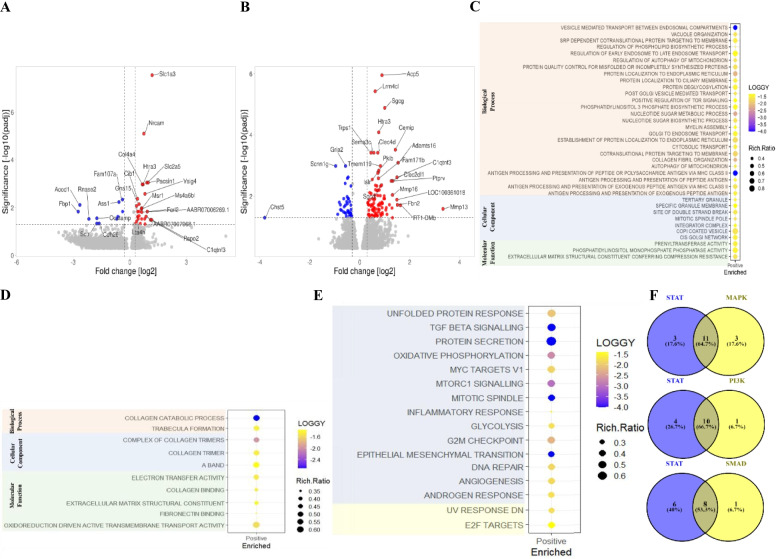
Differential gene expression and gene set enrichment analysis of OA macrophages. Volcano plots showing the top differentially expressed genes of macrophages at 4-weeks (**A**) and 12-weeks (**B**) post surgery, as compared to sham controls. The Y-axis represents the -log10 of the adjusted p-value with a cut-off set at 1.3 (padj < 0.05) and the x-axis represents the log2 fold change with cut-off at -0.5 and 0.5. Bubble plots showing gene set enrichment of Gene Ontology terms at 4-weeks (**C**) and 12-weeks (**D**) and enrichment of Hallmark term at 4-weeks (**E**) after OA induction versus sham controls. LOGGY represents the -log10 of the false discovery rate (FDR <0.05) and Rich.Ratio the number of genes called to a set divided by the total number of genes in the set. Venn diagrams comparing the number significantly enriched hallmark pathways in early-stage OA development (shown in panel E) that are associated with STAT signaling and their overlap with pathways involving MAPK, PI3K, and SMAD signaling mechanisms (**F**)

Forty-two genes were differentially expressed in synovial macrophages at 4-weeks after OA induction (18 increased and 24 decreased) (Sup. 1). These included genes involved in cellular metabolism (*Fbp1, Acod1, C1qtnf3, Slc1A3)*, macrophage activation (*Rnase2, Ocstamp*), inflammation (*Sctr*), cell motility (*Cdh26*), and Wnt signalling (*Rspo2)* (Fig. 1A). One hundred thirty-three genes were differentially expressed in synovial macrophages at 12-weeks (78 increased and 55 decreased) (Sup. 2). These included genes involved in extracellular remodelling (*Mmp16, Fbn2, Adamts16, Mmp13, Vit, Cemip*), cell motility (*Myh7, Tnni1*), and cell fate (*Ptprv*) (Fig. 1B).

Gene ontology analysis identified 46 gene sets enriched at 4 weeks (OA vs sham) (Sup. 3), including cellular metabolism, cellular stress, protein secretion, and macrophage function (Fig. 1C). At 12-weeks post-surgery (OA vs sham), enriched gene sets were primarily extracellular matrix remodeling (Fig. 1D, Sup. 4). At 4 weeks post-OA induction, synovial macrophage activation was associated with 16 enriched Hallmark pathways (Fig. 1E, Sup. 5). Of these, 14 pathways involved STAT, 14 involved mitogen activated protein kinase (MAPK), 11 involved phosphoinositide 3-kinase (PI3K), and 9 involved SMAD signaling (Fig. 1E, Sup. 5). STAT signaling-related pathways overlapped substantially with MAPK (64.7%), PI3K (66.7%), and SMAD (53.3%) signaling (Fig. 1F).

### Mechanical pain sensitivity

#### Pressure application measurement at the knee

**Fig. 2:**
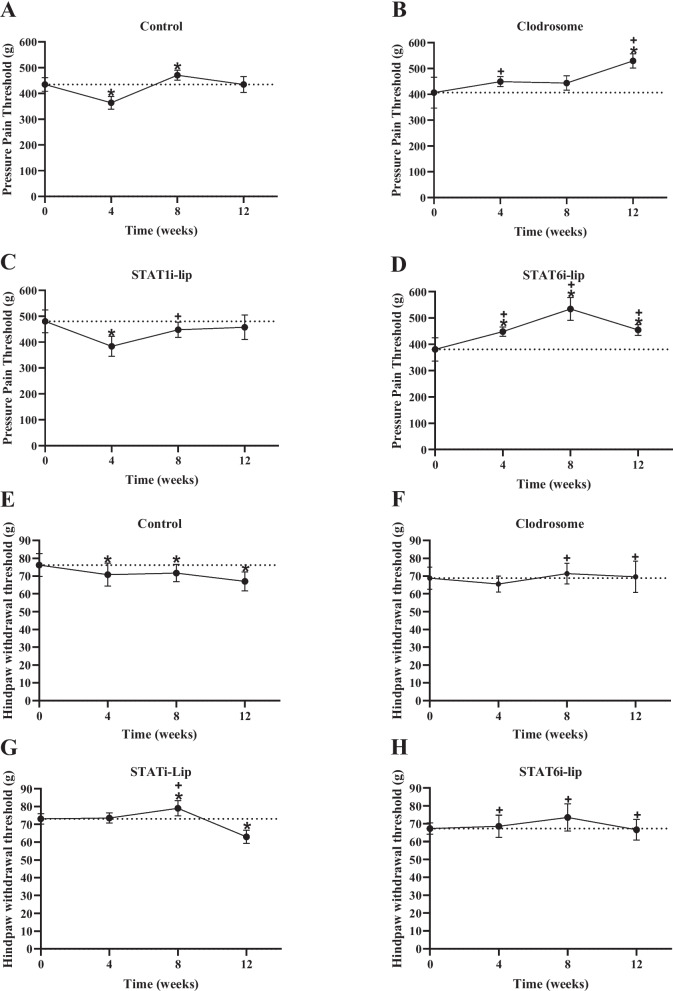
Knee pressure pain and hindpaw withdrawal thresholds. Pressure pain threshold as measured by pressure application measurement of Veh-lip (control) (**A**), Clod-lip (**B**), STAT1i-lip (**C**), and STAT6i-lip (**D**) treatment at pre-surgical baseline (0), and 4-, 8- and 12-weeks post OA induction. Hindpaw withdrawal threshold as measured by electronic von Frey of Veh-lip (control) (**E**), Clod-lip (**F**), STAT1i-lip (**G**), and STAT6i-lip (**H**) treatment at pre-surgical baseline (0), and 4-, 8- and 12-weeks post OA induction. The y-axis represents force (grams, g) applied to the hindpaw before withdrawal and the x-axis time in weeks. Mean with 95% confidence intervals are displayed. Significant differences versus baseline (*) and versus control (Veh-lip) (+) are shown (P <0.05)

Compared to baseline (pre-surgery) measures, the PAM threshold decreased (more pain sensitivity) at 4- (β coefficient [95% confidence interval]) (-70.57g [-102.37, -38.77]), increased at 8- (36.42g [4.62, 68.22]), and returned to baseline at 12-weeks (0.37g [-31.43, 32.17]) after OA induction surgery and control liposome (Veh-lip) injections (Fig. 2A, Sup. 6A).

Macrophage depletion using clodronate-laden liposomes (Clod-lip) prevented a decrease in the PAM threshold at 4- (42.18g [-3.27, 87.63]), and increased (improved) the threshold at 12-weeks (123.32g [77.87, 168.77]) compared to baseline (Fig. 2B, Sup. 6B). Compared to Veh-lip controls, Clod-lip increased the PAM threshold at 4- (112.75g [50.60, 174.90]) and 12-weeks (122.95g [60.80, 185.10]) (Fig. 2B, Sup. 7).

STAT1-inhibitor liposomes (STAT1i-lip) did not prevent a decrease in the PAM threshold at 4- (-96.64g [-146.19, -47.09]), and prevented an increase in the threshold at 8-weeks (-32.44g [-81.99, 17.11]) compared to baseline (Fig. 2C, Sup. 6C). Compared to Veh-lip controls, STAT1i-lip decreased the PAM threshold at 8-weeks (-68.86g [-131.01, -6.71]) (Fig. 2C, Sup. 7).

STAT6-inhibitor liposomes (STAT6i-lip) increased the PAM threshold at 4- (67.86g [6.38, 109.34]), 8- (153.85g [112.37, 195.33]), and 12-weeks (73.97g [32.49, 115.45]) compared to baseline (Fig. 2D, Sup 6D). Compared to Veh-lip controls, STAT6i-lip increased the withdrawal threshold at 4- (138.43g [76.28, 200.58]), 8- (117.43g [55.28, 179.58]), and 12-weeks (73.60g [11.45, 135.75]) (Fig. 2D, Sup. 7).

#### Hindpaw withdrawal threshold

Compared to baseline measures, the hindpaw withdrawal threshold decreased at 4- (-9.18g [-17.18, -1.18]), 8- (-8.23g [-16.23, -0.022]), and 12-weeks (-12.93g [-20.93, -4.93]) after OA induction surgery and Veh-lip injections (Fig. 2E, Sup. 8A).

Clod-lip injections prevented reductions in hindpaw withdrawal threshold at all time points compared to baseline. Compared to Veh-lip controls, Clod-lip injections increased the withdrawal threshold at 8- (10.83g [0.8, 20.85]), and 12-weeks (13.75g [3.73, 23.79]) (Fig. 2F, Sup. 9).

STAT1i-lip prevented a decrease in hindpaw withdrawal threshold at 4-, increased the threshold at 8- (5.89g [1.71, 10.07]), but did not prevent a decrease in the withdrawal threshold at 12-weeks (-10.16g [-14.34, -5.98]) compared to baseline (Fig. 2G, Sup. 8C). Compared to Veh-lip controls, STAT1i-lip increased withdrawal threshold only at 8-weeks (14.12g [4.09, 24.14]).

STAT6i-lip injections prevented decreases in withdrawal threshold at all time points compared to baseline. Compared to Veh-lip controls, STAT6i-lip increased withdrawal thresholds at 4- (10.45g [0.42, 20.47]), 8- (14.43g [4.40, 24.46]), and 12-weeks (12.26g [2.23, 22.28]) (Fig. 2H, Sup.9).

## Histopathology

### Synovial histopathology

**Fig. 3:**
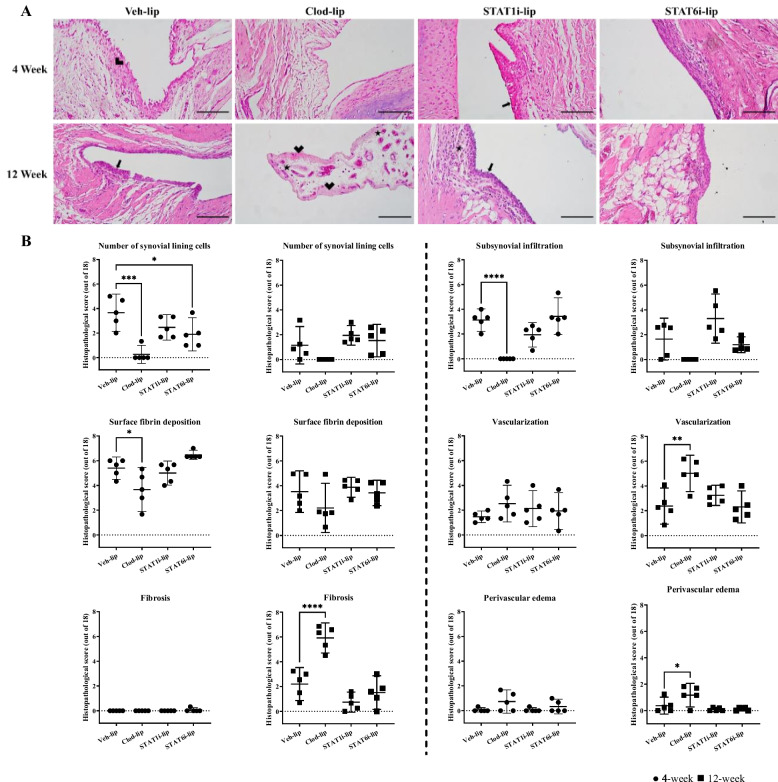
Synovial histopathology and synovitis grading during experimental OA development. Representative images of H&E-stained synovium at 4- and 12-weeks post surgery (**A**). The scale bar represents 100μm, the thick arrow; synovial hyperplasia, thin arrow; sub synovial infiltrate, star; angiogenesis, and arrowhead; synovial fibrosis. Individual measures of synovial histopathology at 4-weeks (●) and 12-weeks (◼) post surgery with comparisons to Veh-lip. The y-axis shows the total histopathological score (out of 18) and the x-axis the treatment group. Mean with 95% confidence intervals are displayed, **** p<0.0001, *** p<0.001, ** p<0.01, * p<0.05

Compared to Veh-lip controls, macrophage depletion (Clod-lip) decreased synovial lining thickness, sub-intimal infiltration, and fibrin deposition at 4-weeks (Fig. 3B), but increased synovial vascularization, perivascular edema, and fibrosis at 12-weeks post-OA induction (Fig. 3C). No changes in synovial histopathology measures were observed with STAT1 inhibition at either time point. STAT6 inhibition (STAT6i-lip) decreased synovial lining thickness at 4-weeks post-OA induction.

### Cartilage histopathology

**Fig. 4:**
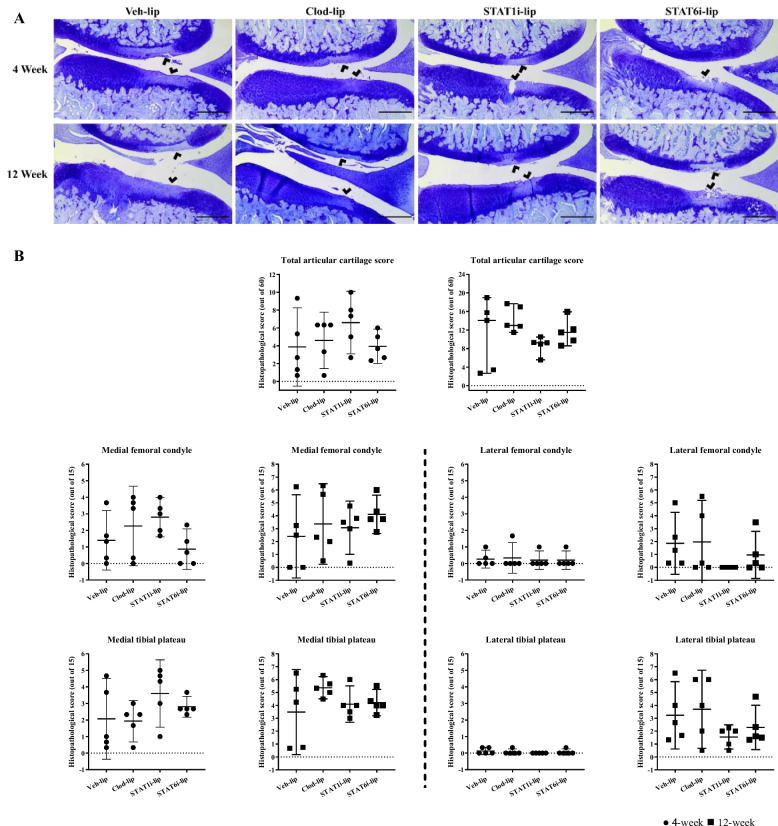
Cartilage histopathology and degeneration grading during experimental OA development. Representative images of toluidine blue stained cartilage at 4- and 12-weeks post surgery (**A**). The scale bar represents 500μm, and the arrowhead areas of cartilage degeneration. Measures of cartilage degeneration in the medial and lateral tibial plateau and femoral condyles at 4-weeks (●) and 12-weeks (◼) post surgery. The y-axis shows the total histopathological score (out of 60) or individual histopathological score (out of 15) and the x-axis the treatment group. Mean with 95% confidence intervals are displayed

Proteoglycan loss, fissuring, and partial thickness cartilage erosion was focused in the central regions of articular surfaces at levels consistent with previous literature, with progression in severity from 4- to 12-week time points (Fig. 4A) [16]. There were no changes in cartilage degeneration scores with any treatment, although a trend toward a decrease in total articular degeneration score was observed at 12-weeks post-OA induction in the STAT1i-lip treatment group in particular (Fig. 4B).

### Immunofluorescence detection of CD68+ macrophages in synovial tissue

**Fig. 5:**
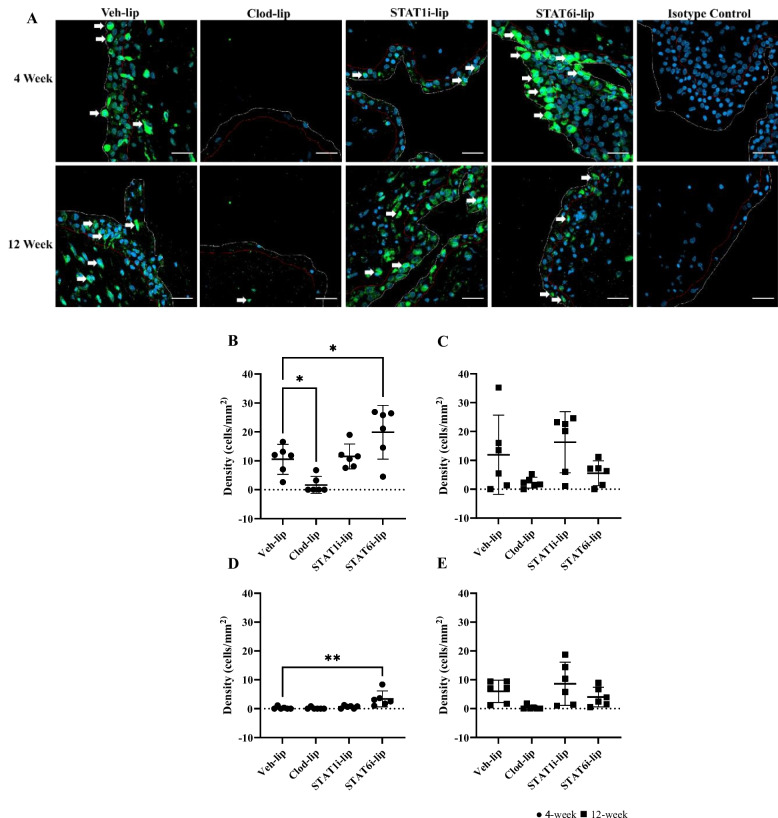
Macrophage quantification in the synovial intima and sub-intima. Representative confocal images of anti-CD68 stained (green) macrophages at 4- and 12-weeks post surgery (**A**). The scale bar represents 25μm. The white line separates the edge of the synovium and joint space and red line the interface between intima and subintima. White arrows highlight CD68 positive cells. Cell density in the intima 4- (●) (**B**) and 12-weeks (◼) (**C**) and subintima 4- (●) (**D**) and 12-weeks (◼) (**E**) post surgery with comparisons to Veh-lip. Mean with 95% confidence intervals are displayed, ** p<0.01, * p<0.05

Intimal macrophage density remained constant whereas subintimal macrophage density increased at 12-weeks in the Veh-lip condition (Fig 5D, E). As expected, clod-lip treatment ablated intimal macrophages 4-weeks post-surgery vs Veh-lip (cells/mm^2^) (1.7 and 10.5 respectively; p=0.03) (Fig 5B). STAT1i-lip treatment caused no detectable effect on macrophage density in the intima. STAT6i-lip increased macrophages in the intima compared to Veh-lip (19.9 and 10.5 respectively; p=0.03) 4-weeks after OA induction (Fig 5B). STAT6i-lip also increased subintimal macrophage density 4-weeks after OA induction vs Veh-lip (3.4 and 0.3 respectively, p=0.002) (Fig 5D). No other treatment caused a detectable effect on subintimal macrophage density.

### Sulfated glycosaminoglycan production and gene expression in articular chondrocytes co-cultured with synovial tissue

**Fig. 6:**
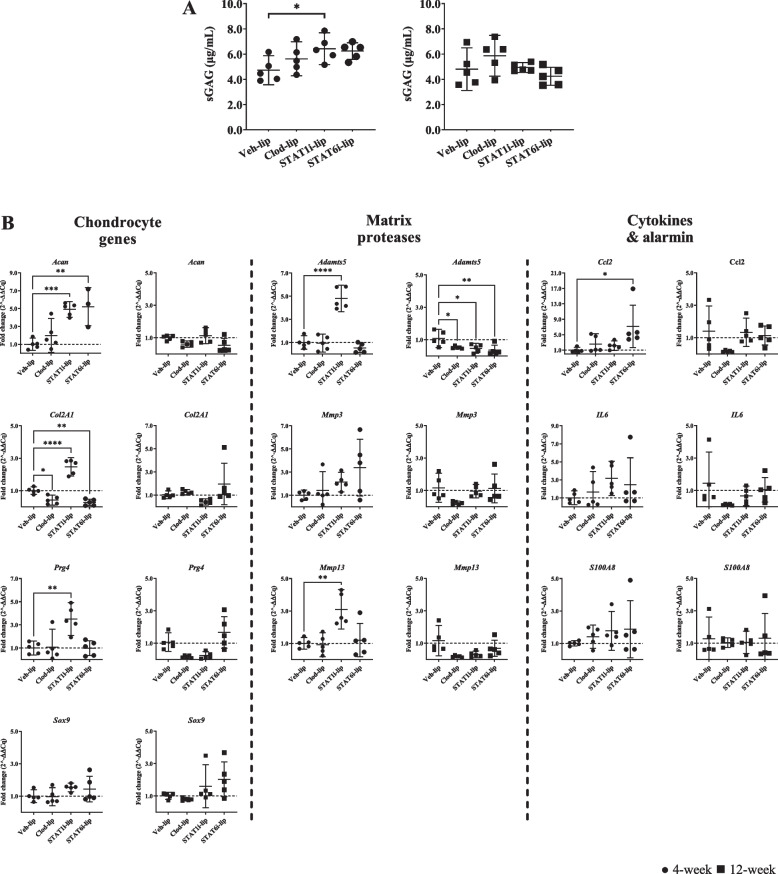
Effects of macrophage-targeted treatment on co-cultured articular chondrocyte sGAG secretion and gene expression. sGAG production by chondrocytes after co-culture with synovial tissue explants from experimental OA knees treated with liposomal treatments for 4- (●) and 12-weeks (◼) (**A**). The Y-axis represents concentration (μg/mL) of sGAGs in media collected after 24 hours of co-culture, * p=0.03. Gene expression of naïve chondrocytes co-cultured with synovial explants from 4- (●) and 12-week (◼) animals (**B**). The y-axis represents fold change (2^-ΔΔCq) and the x-axis treatment group. Mean with 95% confidence intervals are displayed, **** p<0.0001, *** p<0.001, ** p<0.01, * p<0.05

Sulphated glycosaminoglycan (sGAG) production by healthy articular chondrocytes increased in co-culture with STAT1i-lip-treated synovial tissue (compared to Veh-lip) at the 4-week time point (μg/mL) (6.43 and 4.72, respectively; p=0.03) (Fig. 6A). A trend toward increased sGAG secretion was observed in co-culture with STAT6i-lip-treated synovium from the same time point (Fig. 6A). No differences in sGAG secretion were seen when chondrocytes were co-cultured with synovial tissues from the 12-week time point.

Most chondrocyte gene expression changes occurred in response to synovial tissues collected from the 4-week time point. Compared to Veh-lip controls, Clod-lip synovium decreased *Col2a1* expression (fold change) (0.4; p=0.02) in chondrocytes (Fig. 6C). STAT1i-lip synovium increased *Acan* (4.9; p=0.0006), *Col2a1* (2.5; p<0.0001), *Prg4* (3.5; p=0.002), *Adamts5* (4.8; p<0.0001), and *Mmp13* (3.1; p=0.002) expression (Fig. 6C). STAT6i-lip synovium increased A*can* (5.2; p=0.001) and *Ccl2* (7.2; p=0.01), and decreased *Col2a1* (0.3; p=0.003) expression (Fig. 6C). Using synovial tissue from the 12-week time point, Clod-lip, STAT1i-lip, and STAT6i-lip treatment decreased *Adamts5* expression (0.5, 0.4, and 0.5 respectively; p<0.03) in chondrocytes compared to Veh-lip controls (Fig. 6D).

## Discussion

Chronic knee pain is the most commonly reported symptom by patients suffering from OA, but existing treatments are limited by adverse effects [31–33]. Depletion of macrophages can resolve experimental OA related pain [34, 35], but this comes at the cost of increased inflammation and joint tissue damage [12, 13]. This suggested to us that tuning macrophage activation may be more therapeutically effective than ablating macrophages completely. Using transcriptomics, we identified STAT signaling as a dominant intracellular mechanism associated with macrophage activation pathways in early-stage experimental knee OA. Strikingly, STAT6 inhibition (STAT6i) in synovial macrophages raised (improved) the threshold for mechanical sensitivity above baseline and control levels at the affected knee and distal hindpaw at all time points. This robust protection against mechanical pain sensitivity was achieved without worsening synovial or cartilage histopathology outcomes, and reduced synovial lining hyperplasia. In contrast, STAT1 inhibition (STAT1i) transiently lowered (worsened) knee withdrawal thresholds and increased hindpaw withdrawal thresholds, with no clear impact on synovial or cartilage histopathology aside from a trend toward improved cartilage damage at the 12-week endpoint. Lastly, we found that repeated intra-articular injection of clodronate liposomes to persistently deplete synovial macrophages prevented the development of mechanical pain sensitivity up to 12 weeks after OA induction. As expected, these benefits came at the cost of increased joint tissue damage including increased synovial fibrosis, vascularization, and perivascular edema. These results suggest that activated synovial macrophages mediate mechanical pain sensitivity in knee OA and that STAT6 may be a particularly important mechanism.

Macrophages are one of the most important synovial cell types in OA. Although studies have identified altered macrophage-related gene expression from whole synovial tissue [36], our study is among the first to explore differential gene expression selectively within the synovial macrophage compartment during experimental knee OA development. In line with longitudinal gene expression studies in mouse models [37], synovial macrophage gene expression followed a phasic pattern. Early-stage OA macrophage gene expression reflected changes in cellular metabolism, activation, motility, inflammation, and Wnt signaling, which transitioned to extracellular matrix remodeling after 12 weeks of OA development. Similar to transcriptomic analyses in an equine model of OA [38], our gene set enrichment analyses suggested a major role for STAT signaling in mediating cell stress, metabolism, and angiogenesis, with major overlaps across MAPK, PI3K, and SMAD signaling.

A role for STAT signaling in OA cartilage has been described. *Latourte et al.* demonstrated structural protection against joint destabilization-induced experimental knee OA in the mouse using prophylactic systemic inhibition of STAT3 [39]. However, the effects of STAT inhibition on OA-related pain are not well-understood, and macrophage activation mechanisms are context-dependent [40]. Given the complementary roles of STAT1 and STAT6 signaling in regulating macrophage activation, we chose highly selective inhibitors of STAT1 (fludarabine) and STAT6 (AS1517499) for targeted delivery to synovial macrophages via phagocytosis of drug-loaded liposomes.

The potential for dual roles played by macrophages in mediating OA outcomes has long been suspected, partly based on the well-described model of pro- (M1) and anti-inflammatory (M2) polarization. However, our data underscore the hazards of relying on the M1/M2 paradigm, and assuming that all inflammation is bad. For example, interleukin-4 (IL-4) is an anti-inflammatory cytokine and generally regarded as analgesic [41]. IL-4 knock-out mice display increased hindpaw mechanical hypersensitivity and STAT6 is a mediator of IL-4 receptor signaling [40, 42]. Surprisingly, we found that STAT6i robustly protected against the development of both distal and local pain, suggesting that STAT6 likely has alternative functions in OA synovial macrophages. Interestingly, Haraden *et al.* reported that OA disease severity was correlated to synovial fluid levels of the M2-marker CD163 [43], which aligns with our results and suggests that alternatively-activated macrophages may contribute to nociception. Additionally, atopic diseases characterised by STAT6 activation are associated with increased risk of OA [44]. Based on the literature, we predicted that STAT1 would drive pro-inflammatory macrophages and nociception in OA. However, we only observed a brief benefit on distal pain, and worsening of local knee pain sensitivity with STAT1i. Thus, STAT1 signaling may only play a small role in mediating OA-related nociception.

Analgesic treatments are frequently associated with off-target effects. For example, NGF inhibition caused a rare but clinically-important increased risk of rapidly progressive OA [1]. To ensure that macrophage-targeted treatments did not worsen joint tissue outcomes, we explored joint histopathology and crosstalk between treated synovial tissue and healthy primary articular chondrocytes in an *ex-vivo* joint co-culture system [16]. Importantly, no treatment led to any increase in articular cartilage damage. There were trends toward reduced cartilage damage with STAT1i and STAT6i, but we lacked statistical power to detect a small protective effect. We therefore cannot exclude the potential for a protective effect on joint damage, as Sun *et al.* reported marked reduction in cartilage degeneration after depleting macrophages in obese mice prior to, and 1 week after, OA induction [45].

We previously reported that synovial tissue from early-stage experimental knee OA co-cultured with healthy articular chondrocytes stimulates a transient anabolic response [16]. In this study, we found that macrophage-depleted synovium from early-stage OA decreased *Col2a1* expression by healthy articular chondrocytes, suggesting that synovial macrophages control chondrocyte extracellular matrix gene expression through crosstalk mechanisms. Supporting this, synovial tissues treated with liposomal STAT1i caused increased sGAG secretion and expression of matrix (*Acan*, *Col2a1*, and *Prg4*) and protease genes (*Adamts5* and *Mmp13*), whereas STAT6i led to increased *Acan* and *Ccl2,* and decreased *Col2a1* gene expression. Overall, our *in vitro* crosstalk experiments suggest that STAT1-mediated macrophage activation inhibits anabolic responses in chondrocytes, whereas STAT6-mediated macrophage activation may be more important for nociception with fewer effects on chondrocyte anabolism. Given these somewhat complementary findings, it may have been interesting to include a combined STAT1i-STAT6i treatment.

Synovial tissue function is key to maintaining joint homeostasis and we found that synovial macrophage depletion caused worse synovial vascularization, fibrosis, and perivascular edema, which was not seen with STAT1i or STAT6i. Similarly, other studies have found that transient and/or prophylactic depletion of macrophages resulted in worse synovitis, joint damage, and infiltration of T lymphocytes [12, 13]. In those studies, depletion was performed at a single timepoint, whereas we used repeat dosing every 2 weeks to sustain macrophage depletion/suppression. Together with our findings, a clearer picture is emerging that macrophages are essential for maintaining joint homeostasis, while simultaneously playing pathological roles in nociception and engaging in crosstalk with other tissues.

Our study has limitations. Our surgically induced joint destabilization model of OA does not address other OA risk factors such as age, obesity, or female sex, and the effects of STAT inhibition should be confirmed in those settings. Although our study focused on two commonly used methods to assess mechanical pain sensitivity at the knee and hindpaw, other pain-related behavioural tests may have revealed different outcomes. We cannot rule out a role played by other phagocytes such as dendritic cells, mast cells, or neutrophils (rarely seen in OA), which may also have been targeted by liposomes. However, synovial macrophages are the dominant immune cells in healthy joints and are heavily recruited to the synovium of OA joints. Further studies will be required to determine whether different roles are played by tissue resident versus recruited (bone marrow-derived) synovial macrophages.

## Conclusions

Our findings demonstrate that synovial macrophages play a major role in mediating mechanical pain sensitivity in experimental knee OA. Although depleting macrophages improved pain sensitivity, this came at the cost of increased joint tissue damage. Macrophages therefore likely play dual roles in pain and joint organ homeostasis, suggesting that inhibiting macrophage activation may be a better treatment strategy than depletion. Selective targeting of macrophages with STAT inhibitors is a novel candidate strategy for pain modification in OA, and STAT6i confers substantial protection against mechanical pain sensitivity without aggravating synovial histopathology. Further studies are warranted to assess the effects of this treatment approach in other OA contexts.

### Supplementary Information

**Supplementary material 1.**

**Supplementary material 2.**

## Data Availability

RNA sequencing data files were deposited in the Gene Expression Omnibus (GEO) at the NCBI (GSE216932). All other datasets used for the present study are available from the corresponding author upon reasonable request

## Abbreviations

OA: Osteoarthritis
NGF: Nerve growth factor
STAT: Signal transducer and activator of transcription
Veh-lip: Phosphate buffered saline (vehicle) containing liposome
Clod-lip: Chlodronate containing liposome
STAT1i: STAT1 inhibition
STAT1i-lip: STAT1 inhibitor containing liposome
STAT6i: STAT6 inhibition
STAT6i-lip: STAT6 inhibitor containing liposome
PAM: Pressure application Measurement
eVF: Electronic von Frey
H&E: Haematoxylin and eosin
sGAG: Sulfated glycosaminoglycan
MAPK: Mitogen activated protein kinase
PI3K: Phosphoinositide 3-kinase
M1: Proinflammatory macrophage
M2: Anti-inflammatory macrophage
IL-4: Interleukin-4
