# Supplementary material for: Targeting STAT6-mediated synovial macrophage activation improves pain in experimental knee osteoarthritis
Source: Arthritis Res Ther. 2024 Mar 20;26:73. doi: 10.1186/s13075-024-03309-6 (PMC10953260; doi:10.1186/s13075-024-03309-6)
Supplement: Supplementary file 1 — Supplementary material 1. [file 13075_2024_3309_MOESM1_ESM.pdf]

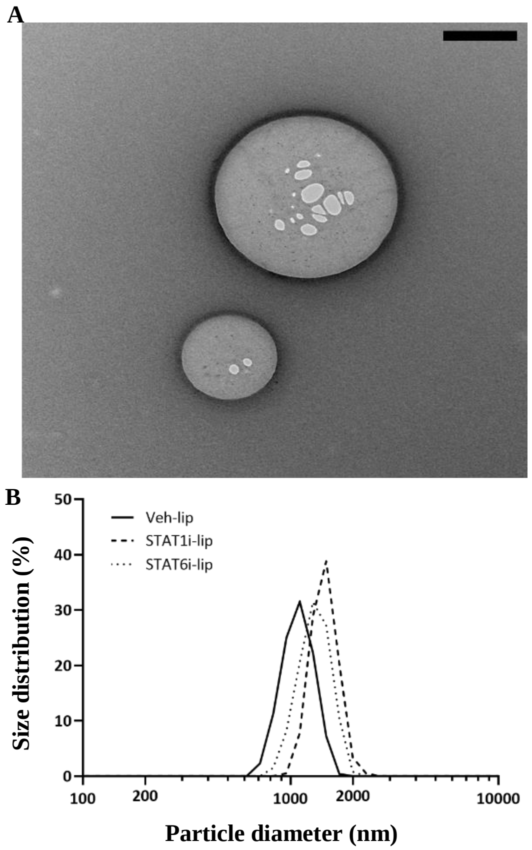

Supplemental Fig 1. Liposome shape and size distribution. Representative photomicrograph of prepared drug containing liposome **(A)**. Scale bar represents 0.5 µm. Representative dynamic light scattering results are displayed **(B)** with the y-axis showing volume distribution (% of total population) and x-axis the particle diameter.
