# Supplementary material for: Targeting STAT6-mediated synovial macrophage activation improves pain in experimental knee osteoarthritis
Source: Arthritis Res Ther. 2024 Mar 20;26:73. doi: 10.1186/s13075-024-03309-6 (PMC10953260; doi:10.1186/s13075-024-03309-6)
Supplement: Supplementary file 2 — Supplementary material 2. [file 13075_2024_3309_MOESM2_ESM.pdf]

Supplemental table 1. Differentially expressed genes at 4-weeks. Differentially expressed genes with an adjusted p value (padj) <0.05. Genes upregulated in OA are represented by a positive fold change (Log2FoldChange) and genes downregulated by a negative fold change.

Row.names	log2FoldChange	padj	external_gene_name
ENSRNOG00000016163	1.180428996	2.82E-08	Slc1a3
ENSRNOG00000004067	0.745566281	7.99E-06	Nrcam
ENSRNOG00000017693	0.902116131	0.000876432	Slc2a5
ENSRNOG00000054603	0.970000223	0.000876432	Pacsin1
ENSRNOG00000014851	0.661965165	0.001037881	Col4a4
ENSRNOG00000008182	0.70338452	0.001037881	Htra3
ENSRNOG00000038132	0.803639935	0.002615602	Vsig4
ENSRNOG00000033498	0.405260428	0.004188724	Cib1
ENSRNOG00000033261	-0.390366395	0.004494457	Fam107a
ENSRNOG00000008837	-0.577310749	0.005753927	Ass1
ENSRNOG00000005378	0.486977437	0.007076163	Gna15
ENSRNOG00000009919	-2.648723536	0.007585942	Acod1
ENSRNOG00000004494	0.355373947	0.008374958	Lta4h
ENSRNOG00000012779	0.632039589	0.01019025	Msr1
ENSRNOG00000030689	0.617601711	0.011195323	Ms4a6bl
ENSRNOG00000061524	0.490009107	0.014419012	AABR07068285.2
ENSRNOG00000051487	0.414310325	0.014419012	Kremen1
ENSRNOG00000017597	-2.737601371	0.014419012	Fbp1
ENSRNOG00000015948	-0.386477118	0.014419012	Slc1a5
ENSRNOG00000016164	0.925455677	0.014419012	Fcrl2
ENSRNOG00000018566	0.212444568	0.015166772	Ctsl
ENSRNOG00000047349	0.629392319	0.015166772	AABR07006269.1
ENSRNOG00000009982	0.19842526	0.015166772	Pnp
ENSRNOG00000018400	0.492888159	0.021751535	Golm1
ENSRNOG00000009341	-0.633612016	0.023346679	Hivep3
ENSRNOG00000017438	0.86350558	0.023346679	AABR07007068.1
ENSRNOG00000014475	0.40409469	0.023796129	Slc31a1
ENSRNOG00000019110	-1.745048249	0.027476515	Ocstamp
ENSRNOG00000013578	0.445099695	0.029400882	Trem2
ENSRNOG00000032133	-2.169371231	0.029400882	Rnase2
ENSRNOG00000005679	0.786819688	0.029400882	Fap
ENSRNOG00000026807	0.627138755	0.029400882	LOC362863
ENSRNOG00000056493	-0.691736731	0.029400882	Mybpc1
ENSRNOG00000037687	1.098177278	0.032049809	Rspo2
ENSRNOG00000018570	1.157760966	0.032214387	C1qtnf3
ENSRNOG00000012062	0.360866569	0.036526701	Npc2
ENSRNOG00000053332	-1.628415334	0.045540302	Cdh26
ENSRNOG00000049766	-1.747079821	0.045723635	Sctr
ENSRNOG00000005249	0.245673363	0.045723635	Snx6

ENSRNOG00000000142	0.317245961	0.045723635	Plxdc2
ENSRNOG00000000855	0.557795078	0.045843032	Lst1
ENSRNOG00000014603	0.80904359	0.04858025	Sgcg

Supplemental table 2. Differentially expressed genes at 12-weeks. Differentially expressed genes with an adjusted p value (padj) <0.05. Genes upregulated in OA are represented by a positive fold change (Log2FoldChange) and genes downregulated by a negative fold change.

Row.names	log2FoldChange	padj	external_gene_name
ENSRNOG00000046261	0.890052296	1.11E-06	Acp5
ENSRNOG00000026302	0.621483643	3.72E-06	Lrrn4cl
ENSRNOG00000014603	0.999361928	1.30E-05	Sgcg
ENSRNOG00000008182	0.762441198	8.22E-05	Htra3
ENSRNOG00000012442	1.42799731	0.000306367	Cemip
ENSRNOG00000024998	0.451528157	0.000384099	Trps1
ENSRNOG00000006526	0.56583181	0.000384099	Sema3c
ENSRNOG00000010181	0.727980835	0.000384099	Clec4d
ENSRNOG00000016812	1.538124752	0.000814165	Adamts16
ENSRNOG00000017842	-0.961721711	0.001042466	Scnn1g
ENSRNOG00000054204	-0.566585949	0.001042466	Gria2
ENSRNOG00000000700	0.830366968	0.001042466	Tmem119
ENSRNOG00000000811	0.766698419	0.001464273	Pkib
ENSRNOG00000048924	0.73032045	0.001716486	Islr
ENSRNOG00000013269	-0.461252983	0.00235593	Tnfsf10
ENSRNOG00000018570	1.491432586	0.002378731	C1qtnf3
ENSRNOG00000004783	0.748665156	0.00255907	Fam171b
ENSRNOG00000023303	-0.493034271	0.002560881	Dpep2
ENSRNOG00000005931	0.505377634	0.002786547	Cpq
ENSRNOG00000007866	1.262693056	0.003122938	Clec2dl1
ENSRNOG00000005277	1.321259015	0.003122938	Ptprv
ENSRNOG00000009892	-0.580610529	0.00376258	Adamts15
ENSRNOG00000016372	-0.282071876	0.003915687	Slc12a7
ENSRNOG00000002372	0.874055931	0.004285156	Sgcd
ENSRNOG00000004067	0.630925222	0.004349213	Nrcam
ENSRNOG00000016968	-0.351973032	0.00457206	Gramd4
ENSRNOG00000010977	0.692385055	0.00457206	Igfbp6
ENSRNOG00000017786	0.834954167	0.004631346	Acta1
ENSRNOG00000007461	0.633547014	0.006052383	Klhl41
ENSRNOG00000027936	0.78188318	0.006141339	Ccdc8
ENSRNOG00000030210	0.730591683	0.006371413	Fndc1
ENSRNOG00000006548	0.692459913	0.006502247	Mrc2
ENSRNOG00000010918	0.401409229	0.007540242	Cebpa
ENSRNOG00000009972	-0.267587962	0.008098586	Rara
ENSRNOG00000002345	0.460519623	0.008098586	Rasgef1b
ENSRNOG00000005708	1.310923614	0.008974323	Mmp16
ENSRNOG00000008680	0.600455103	0.009054238	Loxl1
ENSRNOG00000022710	0.722509356	0.009385557	Prrg4
ENSRNOG00000005679	0.824225794	0.009519663	Fap

ENSRNOG00000004583	0.983260413	0.009647513	Mb
ENSRNOG00000051688	-0.502607515	0.010264192	Syt15
ENSRNOG00000030763	-0.638481422	0.011868307	Dpp4
ENSRNOG00000012681	-0.461006623	0.011868307	Lgals9
ENSRNOG00000018669	-0.450891881	0.011868307	Jak3
ENSRNOG00000014398	0.607866224	0.011868307	Scara5
ENSRNOG00000023812	0.965379606	0.011868307	Raver2
ENSRNOG00000010079	0.994431206	0.011868307	Ca3
ENSRNOG00000006052	0.507512071	0.011908438	Sulf2
ENSRNOG00000046494	1.488478451	0.012830497	LOC100361018
ENSRNOG00000009822	0.388653991	0.013189148	Tlr2
ENSRNOG00000021155	0.57562951	0.013189148	Ctsk
ENSRNOG00000030981	-0.521647457	0.014877556	Scnn1b
ENSRNOG00000053201	-0.398984485	0.014877556	Gpcpd1
ENSRNOG00000007545	-0.817073782	0.015648813	Angptl4
ENSRNOG00000013631	0.329871237	0.015648813	Slc31a2
ENSRNOG00000010666	0.884469294	0.015648813	Ccn5
ENSRNOG00000032297	0.323017785	0.016622334	Msmo1
ENSRNOG00000024620	0.562246532	0.016843063	Mamdc2
ENSRNOG00000018509	0.803547745	0.016843063	Cx3cr1
ENSRNOG00000033496	0.901376684	0.016843063	Igdcc4
ENSRNOG00000058329	0.911675704	0.016843063	Prrx2
ENSRNOG00000009079	-0.495296703	0.017365334	Prkar2b
ENSRNOG00000004400	-0.773219157	0.018797022	Avpr1a
ENSRNOG00000006787	0.350201837	0.018797022	Dhcr24
ENSRNOG00000000902	0.363357584	0.018797022	Hsph1
ENSRNOG00000049471	0.466777684	0.018797022	Steap3
ENSRNOG00000014166	0.758474523	0.018797022	Smoc2
ENSRNOG00000009343	1.191133669	0.018797022	Evpl
ENSRNOG00000043219	1.508025779	0.018871909	Fbn2
ENSRNOG00000047545	-0.570738111	0.019397831	Adra2a
ENSRNOG00000004672	-0.353325583	0.019397831	Sec14l2
ENSRNOG00000008749	0.5100935	0.019397831	Col5a1
ENSRNOG00000012196	0.740907822	0.019397831	Asah2
ENSRNOG00000020743	0.821799203	0.019397831	Cyp2s1
ENSRNOG00000049491	1.631670496	0.019566261	RT1-DMb
ENSRNOG00000057855	0.452330651	0.020988374	F5
ENSRNOG00000049994	-0.619010451	0.021769497	Ifi44l
ENSRNOG00000037838	0.563778448	0.023524739	RGD1560455
ENSRNOG00000039759	0.578150836	0.023524739	Gpr34
ENSRNOG00000000563	0.897097058	0.023524739	Adamts14
ENSRNOG00000016983	1.063372471	0.023524739	Myh7
ENSRNOG00000015972	1.292122146	0.024079731	Ano5
ENSRNOG00000008478	3.33248879	0.025744708	Mmp13

ENSRNOG00000050996	0.648347864	0.026128682	Kctd4
ENSRNOG00000034139	0.746484637	0.026128682	Lyc2
ENSRNOG00000014243	-0.349182886	0.02686541	Pear1
ENSRNOG00000013312	0.508594673	0.027085163	Kcnt2
ENSRNOG00000014505	-0.768625373	0.027244864	Pmfbp1
ENSRNOG00000004712	0.782565835	0.027250161	Angptl1
ENSRNOG00000004706	1.047587761	0.027821961	Vit
ENSRNOG00000061394	0.555255558	0.028158956	Clec4e
ENSRNOG00000017819	0.371624718	0.028312868	Cd14
ENSRNOG00000007036	0.517639602	0.028312868	Tgfbr1
ENSRNOG00000059507	-0.728965502	0.028512068	Lamc3
ENSRNOG00000008351	-0.476449962	0.029518787	RGD1308117
ENSRNOG00000019141	-0.452501871	0.030827837	Ch25h
ENSRNOG00000000653	-0.649222819	0.032074036	Nr5a2
ENSRNOG00000007918	0.587311781	0.032074036	Tbxas1
ENSRNOG00000015505	0.755290855	0.032074036	Mfap5
ENSRNOG00000007286	-0.441207605	0.033802817	Mdm1
ENSRNOG00000052880	-0.593995077	0.034327848	Prph
ENSRNOG00000034258	0.905499984	0.034327848	Xirp2
ENSRNOG00000007567	-0.429052156	0.034650884	Rem1
ENSRNOG00000003229	-0.366534807	0.034650884	Tspan7
ENSRNOG00000011659	0.662982576	0.035201619	Alpk3
ENSRNOG00000061845	-0.548642643	0.035251113	Cttnbp2
ENSRNOG00000004273	-0.353401237	0.036880784	Ifitm1
ENSRNOG00000023148	0.839783456	0.037194877	Col11a1
ENSRNOG00000019757	-0.879158155	0.03860483	Dpep3
ENSRNOG00000039278	0.909031651	0.03860483	Mcart1
ENSRNOG00000043167	0.341170162	0.038812981	Itga9
ENSRNOG00000006867	0.37704266	0.038812981	Etv1
ENSRNOG00000054251	0.630452687	0.038907975	Clec7a
ENSRNOG00000009073	1.093680463	0.038907975	Tnni1
ENSRNOG00000025245	0.490228618	0.040783376	RGD1311946
ENSRNOG00000013663	0.689243577	0.042481784	Tmem86a
ENSRNOG00000014117	-0.341674826	0.042576729	Hmox1
ENSRNOG00000006859	0.365525243	0.042576729	Insig1
ENSRNOG00000017459	0.631539588	0.042576729	C1ql3
ENSRNOG00000002434	0.720128473	0.042576729	Tmem100
ENSRNOG00000032994	0.852028342	0.042576729	Myom3
ENSRNOG00000013179	-0.412391059	0.043321885	Tinagl1
ENSRNOG00000056118	-0.319453441	0.043321885	Cic
ENSRNOG00000038202	0.462908035	0.043515363	Calml4
ENSRNOG00000026604	0.522008395	0.043515363	Cercam
ENSRNOG00000022256	-0.537374547	0.043998835	Cxcl10
ENSRNOG00000013578	0.555124503	0.044615422	Trem2

ENSRNOG00000005592	0.581016282	0.044829054	Brinp2
ENSRNOG00000022597	-0.390437232	0.045006764	Cenpj
ENSRNOG00000019556	0.502988502	0.045006764	Cd9
ENSRNOG00000008622	0.542822201	0.045006764	Creb5
ENSRNOG00000013967	0.504059827	0.047724369	Blnk
ENSRNOG00000021521	-3.801777205	0.050067516	Chst5

Supplemental table 3. GSEA report for GO at 4-week. Gene sets positively enriched in PTOA from the Gene Ontology database at 4-weeks.

NAME	NES	NOM p-val	FDR q-val
GOBP_ANTIGEN_PROCESSING_AND_PRESENTATION_OF_PEPTIDE_OR_POLYSACCHARIDE_ANTIGEN_VIA_MHC_CLASS_II	2.527666	0	0.006856685
GOBP_NUCLEOTIDE_SUGAR_METABOLIC_PROCESS	2.468147	0	0.005145371
GOBP_VESICLE_MEDIATED_TRANSPORT_BETWEEN_ENDOSOMAL_COMPARTMENTS	2.4302742	0	0.006869598
GOBP_PROTEIN_LOCALIZATION_TO_ENDOPLASMIC_RETICULUM	2.4022214	0	0.005152199
GOBP_ESTABLISHMENT_OF_PROTEIN_LOCALIZATION_TO_ENDOPLASMIC_RETICULUM	2.3398695	0	0.009028094
GOBP_COTRANSLATIONAL_PROTEIN_TARGETING_TO_MEMBRANE	2.3228097	0	0.01155867
GOBP_POST_GOLGI_VESICLE_MEDIATED_TRANSPORT	2.304336	0	0.013865072
GOCC_MITOTIC_SPINDLE_POLE	2.2978964	0	0.012959702
GOBP_COLLAGEN_FIBRIL_ORGANIZATION	2.2952921	0	0.011519736
GOCC_COPI_COATED_VESICLE	2.2122838	0	0.02528621
GOMF_EXTRACELLULAR_MATRIX_STRUCTURAL_CONSTITUENT_CONFERRING_COMPRESSION_RESISTANCE	2.202142	0	0.025801998
GOBP_AUTOPHAGY_OF_MITOCHONDRION	2.1820586	0	0.029995421
GOBP_VACUOLE_ORGANIZATION	2.1803434	0	0.02768808
GOBP_REGULATION_OF_AUTOPHAGY_OF_MITOCHONDRION	2.1777034	0	0.02692886
GOBP_ANTIGEN_PROCESSING_AND_PRESENTATION_OF_EXOGENOUS_PEPTIDE_ANTIGEN_VIA_MHC_CLASS_II	2.1706033	0	0.028117338
GOBP_CYTOSOLIC_TRANSPORT	2.1526976	0	0.031778224
GOBP_NUCLEOTIDE_SUGAR_BIOSYNTHETIC_PROCESS	2.1395595	0	0.03397925
GOBP_PROTEIN_QUALITY_CONTROL_FOR_MISFOLDED_OR_INCOMPLETELY_SYNTHESIZED_PROTEINS	2.1369963	0	0.032862827
GOCC_SITE_OF_DOUBLE_STRAND_BREAK	2.1286125	0	0.03311641
GOBP_SRP_DEPENDENT_COTRANSLATIONAL_PROTEIN_TARGETING_TO_MEMBRANE	2.1202404	0	0.035254776
GOCC_CIS_GOLGI_NETWORK	2.1195056	0	0.034069378
GOCC_SPECIFIC_GRANULE_MEMBRANE	2.1105418	0	0.034882184
GOBP_LYTIC_VACUOLE_ORGANIZATION	2.1069336	0	0.035458162
GOCC_GOLGI_STACK	2.106341	0	0.034124304
GOBP_REGULATION_OF_EARLY_ENDOSOME_TO_LATE_ENDOSOME_TRANSPORT	2.1035776	0	0.03331029
GOBP_PROTEIN_LOCALIZATION_TO_CILIARY_MEMBRANE	2.098182	0	0.034179416
GOBP_GOLGI_TO_ENDOSOME_TRANSPORT	2.091858	0.005128205	0.03472157
GOMF_PRENYLTRANSFERASE_ACTIVITY	2.0665827	0	0.040544823
GOBP_PROTEIN_LOCALIZATION_TO_MICROTUBULE_ORGANIZING_CENTER	2.0622885	0	0.04121025
GOCC_DYSTROPHIN_ASSOCIATED_GLYCOPROTEIN_COMPLEX	2.054218	0.004854369	0.042858638
GOBP_REGULATION_OF_PHOSPHOLIPID_BIOSYNTHETIC_PROCESS	2.0388882	0	0.04750668
GOBP_ORGANELLE_DISASSEMBLY	2.0355656	0	0.046778537
GOBP_PROTEIN_EXIT_FROM_ENDOPLASMIC_RETICULUM	2.0342562	0	0.045675196
GOBP_ANTIGEN_PROCESSING_AND_PRESENTATION_OF_PEPTIDE_ANTIGEN	2.032697	0	0.044633698
GOBP_ANTIGEN_PROCESSING_AND_PRESENTATION_OF_EXOGENOUS_PEPTIDE_ANTIGEN	2.0310414	0	0.04376037
GOMF_PHOSPHATIDYLINOSITOL_MONOPHOSPHATE_PHOSPHATASE_ACTIVITY	2.0295067	0.004901961	0.043026023
GOBP_REGULATION_OF_ERYTHROCYTE_DIFFERENTIATION	2.0259774	0	0.0430956
GOBP_AMINO_SUGAR_METABOLIC_PROCESS	2.0242229	0	0.042323843
GOCC_COPI_COATED_VESICLE_MEMBRANE	2.0200748	0	0.04320737
GOCC_SPECIFIC_GRANULE	2.0038197	0	0.048485585
GOBP_PROTEIN_N_LINKED_GLYCOSYLATION	1.9997069	0	0.04856924
GOBP_ENDOPLASMIC_RETICULUM_TO_GOLGI_VESICLE_MEDIATED_TRANSPORT	1.9960654	0	0.048977233
GOBP_PROTEIN_LOCALIZATION_TO_CYTOSKELETON	1.9926087	0	0.049449492
GOBP_PROTEIN_DEGLYCOSYLATION	1.9904077	0.005780347	0.048943087
GOBP_MYELIN_ASSEMBLY	1.9891163	0.005291005	0.04833131
GOBP_POSITIVE_REGULATION_OF_GLYCOPROTEIN_METABOLIC_PROCESS	1.9876896	0	0.047952738

Supplemental table 4. GSEA report for GO at 12-week. Gene sets positively enriched in PTOA from the Gene Ontology database at 12-weeks.

Name	NES	NOM p-val	FDR q-val
GOBP_COLLAGEN_CATABOLIC_PROCESS	2.19	0	0.002
GOCC_COMPLEX_OF_COLLAGEN_TRIMERS	2.1	0	0.011
GOMF_ELECTRON_TRANSFER_ACTIVITY	2.03	0	0.034
GOMF_OXIDOREDUCTION_DRIVEN_ACTIVE_TRANSMEMBRANE_TRANSPORTER_ACTIVITY	2.02	0	0.028
GOMF_EXTRACELLULAR_MATRIX_STRUCTURAL_CONSTITUENT_CONFERRING_TENSILE_STRENGTH	2.02	0	0.022
GOMF_COLLAGEN_BINDING	1.98	0	0.036
GOBP_TRABECULA_FORMATION	1.98	0	0.036
GOCC_COLLAGEN_TRIMER	1.97	0	0.036
GOMF_FIBRONECTIN_BINDING	1.95	0	0.041
GOMF_EXTRACELLULAR_MATRIX_STRUCTURAL_CONSTITUENT	1.95	0	0.041
GOCC_A_BAND	1.94	0	0.044
GOMF_PROTON_TRANSMEMBRANE_TRANSPORTER_ACTIVITY	1.92	0	0.05
GOCC_RESPIRASOME	1.92	0	0.047
GOBP_EXTERNAL_ENCAPSULATING_STRUCTURE_ORGANIZATION	1.92	0	0.045
GOBP_ANTIGEN_PROCESSING_AND_PRESENTATION_OF_PEPTIDE_OR_POLYSACCHARIDE_ANTIGEN_VIA_MHC_CLASS_II	1.91	0	0.048

Supplemental table 5. GSEA report for Hallmark at 4-week. Gene sets positively enriched in PTOA from the Hallmark database at 4-weeks.

NAME	NES	NOM p-val	FDR q-val
HALLMARK_PROTEIN_SECRETION	2.7417886	0	0
HALLMARK_TGF_BETA_SIGNALING	2.3160634	0	0
HALLMARK_EPITHELIAL_MESENCHYMAL_TRANSITION	2.2345629	0	0
HALLMARK_MITOTIC_SPINDLE	2.1072602	0	0
HALLMARK_OXIDATIVE_PHOSPHORYLATION	1.9979291	0	0.001599587
HALLMARK_MTORC1_SIGNALING	1.9896821	0	0.001332989
HALLMARK_G2M_CHECKPOINT	1.8814024	0	0.00647452
HALLMARK_UNFOLDED_PROTEIN_RESPONSE	1.7856348	0	0.00866443
HALLMARK_ANGIOGENESIS	1.6482159	0.016129032	0.014726359
HALLMARK_DNA_REPAIR	1.6304616	0	0.013786918
HALLMARK_MYC_TARGETS_V1	1.6286215	0	0.012533561
HALLMARK_GLYCOLYSIS	1.5741733	0	0.018020747
HALLMARK_ANDROGEN_RESPONSE	1.5708091	0	0.01786499
HALLMARK_UV_RESPONSE_DN	1.5558542	0	0.0171602
HALLMARK_INFLAMMATORY_RESPONSE	1.4775454	0	0.026116434
HALLMARK_E2F_TARGETS	1.4163634	0	0.038666215

Supplemental table 6. Individual linear mixed effects models, PAM. Individual linear mixed effects modelling of pressure pain threshold in Veh-lip **(A)**, Clod-lip **(B)**, STAT1i-lip **(C)**, and STAT6i-lip **(D)**.

A

Model 1 PAM Veh-lip

Fixed Effects			
**Variable**	**β coefficient**	**Standard Error**	**95% Confidence Intervals**
**Time (weeks)**			
0	Reference	Reference	Reference
4	**-70.57**	**16.23**	**-102.37 to -38.77**
8	**36.42**	**16.23**	**4.62 to 68.22**
12	0.37	16.23	-31.43 to 32.17
**Intercept**	434.52	11.47	412.03 to 457.01
Random Effect			
**Variable**	**Variance**	**Standard Error**	**95% Confidence Intervals**
Intercept	1316.24	310.24	829.29 to 2089.13

B

Model 2 PAM Clod-lip

Fixed Effects			
**Variable**	**β coefficient**	**Standard Error**	**95% Confidence Intervals**
**Time (weeks)**			
0	Reference	Reference	Reference
4	42.18	23.19	-3.27 to 87.63
8	36.81	23.19	-8.64 to 82.26
12	**123.32**	**23.19**	**77.87 to 168.77**
**Intercept**	406.92	16.40	374.78 to 439.06
Random Effect			
**Variable**	**Variance**	**Standard Error**	**95% Confidence Intervals**
Intercept	2688.65	633.72	1693.96 to 4267.42

C

Model 3 PAM STAT1i-lip

Fixed Effects			
**Variable**	**β coefficient**	**Standard Error**	**95% Confidence Intervals**
**Time (weeks)**			
0	Reference	Reference	Reference
4	**-96.64**	**25.28**	**-146.19 to -47.09**
8	-32.44	25.28	-81.99 to 17.11
12	-22.84	25.28	-72.39 to 26.71
**Intercept**	480.18	17.88	445.14 to 515.22
Random Effect			
**Variable**	**Variance**	**Standard Error**	**95% Confidence Intervals**
Intercept	3196.11	753.33	2013.68 to 5072.85

D

Model 4 PAM STAT6i-lip

Fixed Effects			
**Variable**	**β coefficient**	**Standard Error**	**95% Confidence Intervals**
**Time (weeks)**			
0	Reference	Reference	Reference
4	**67.86**	**21.17**	**26.38 to 109.34**
8	**153.85**	**21.17**	**112.37 to 195.33**
12	**73.97**	**21.17**	**32.49 to 115.45**
**Intercept**	380.6	14.97	351.27 to 409.93
Random Effect			
**Variable**	**Variance**	**Standard Error**	**95% Confidence Intervals**
Intercept	2239.98	527.97	1411.28 to 3555.28

Supplemental table 7. Linear mixed effects model with pairwise comparisons, PAM.

Model 5: PAM

Fixed Effects			
**Variable**	**β coefficient**	**Standard Error**	**95% Confidence Intervals**
**Treatment**			
Veh-Lip	Reference	Reference	Reference
Clod-Lip	-27.60	21.72	-70.18 to 14.98
Stat1i-Lip	**45.66**	**21.72**	**3.08 to 88.24**
Stat6i-Lip	**-53.92**	**21.72**	**-96.50 to -11.34**
**Time (weeks)**			
0	Reference	Reference	Reference
4	**-70.57**	**22.42**	**-114.52 to -26.62**
8	36.42	22.42	-7.53 to 80.37
12	0.37	22.42	-43.58 to 44.32
**Treatment x Time**			
Clod-Lip (4wk)	**112.75**	**31.71**	**50.60 to 174.90**
Clod-Lip (8wk)	0.39	31.71	-61.76 to 62.54
Clod-Lip (12wk)	**122.95**	**31.71**	**60.80 to 185.10**
Stat1i-Lip (4wk)	-26.07	31.71	-88.22 to 36.08
Stat1i-Lip (8wk)	**-68.86**	**31.71**	**-131.01 to -6.71**
Stat1i-Lip (12wk)	-23.21	31.71	-85.36 to 38.94
Stat6i-Lip (4wk)	**138.43**	**31.71**	**76.28 to 200.58**
Stat6i-Lip (8wk)	**117.43**	**31.71**	**55.28 to 179.58**
Stat6i-Lip (12wk)	**73.60**	**31.71**	**11.45 to 135.75**
**Intercept**	434.52	15.36	404.41 to 464.63
Random Effects			
**Variable**	**Variance**	**Standard Error**	**95% Confidence Intervals**
Intercept (all)	2360.24	279.92	1870.71 to 2977.88
Intercept (by group)	-153.50	140.87	-429.61 to 122.60
Exchangeable covariance matrix used to control for repeated measures			

Supplemental table 8. Individual linear mixed effects models, eVF. Individual linear mixed effects modelling of hind paw withdrawal threshold in Veh-lip **(A)**, Clod-lip **(B)**, STAT1i-lip **(C)**, and STAT6i-lip **(D)**.

A

Model 6 eVF Veh-lip			
Fixed Effects			
**Variable**	**β coefficient**	**Standard Error**	**95% Confidence Intervals**
**Time (weeks)**			
0	Reference	Reference	Reference
4	**-9.18**	**4.08**	**-17.18 to -1.18**
8	**-8.23**	**4.08**	**-16.23 to -0.22**
12	**-12.93**	**4.08**	**-20.93 to -4.93**
**Intercept**	79.91	3.12	73.80 to 86.02
Random Effect			
**Variable**	**Variance**	**Standard Error**	**95% Confidence Intervals**
Intercept	83.32	22.68	48.88 to 142.04

B

Model 7 eVF Clod-lip			
Fixed Effects			
**Variable**	**β coefficient**	**Standard Error**	**95% Confidence Intervals**
**Time (weeks)**			
0	Reference	Reference	Reference
4	-3.28	4.08	-11.28 to 4.73
8	2.6	4.08	-5.40 to 10.60
12	0.83	4.08	-7.18 to 8.83
**Intercept**	68.77	2.89	63.11 to 74.43
Random Effect			
**Variable**	**Variance**	**Standard Error**	**95% Confidence Intervals**
Intercept	83.34	19.64	52.51 to 132.27

C

Model 8 eVF STAT1i-lip			
Fixed Effects			
**Variable**	**β coefficient**	**Standard Error**	**95% Confidence Intervals**
**Time (weeks)**			
0	Reference	Reference	Reference
4	0.40	2.13	-3.78 to 4.58
8	**5.89**	**2.13**	**1.71 to 10.07**
12	**-10.16**	**2.13**	**-14.34 to -5.98**
**Intercept**	73.12	1.55	70.06 to 76.15
Random Effect			
**Variable**	**Variance**	**Standard Error**	**95% Confidence Intervals**
Intercept	22.72	6.18	13.33 to 38.73

D

Model 9 eVF STAT6i-lip			
Fixed Effects			
**Variable**	**β coefficient**	**Standard Error**	**95% Confidence Intervals**
**Time (weeks)**			
0	Reference	Reference	Reference
4	1.27	3.45	-5.49 to 8.01
8	6.20	3.45	-0.55 to 12.96
12	-0.68	3.45	-7.43 to 6.07
**Intercept**	67.34	2.63	62.18 to 72.50
Random Effect			
**Variable**	**Variance**	**Standard Error**	**95% Confidence Intervals**
Intercept	59.35	16.15	34.82 to 101.19

Supplemental table 9. Linear mixed effects model with pairwise comparisons, eVF.

Model 10 eVF

Fixed Effects			
**Variable**	**β coefficient**	**Standard Error**	**95% Confidence Intervals**
**Treatment**			
Veh-Lip	Reference	Reference	Reference
Clod-Lip	**-11.14**	**3.70**	**-18.40 to -3.89**
Stat1i-Lip	-6.81	3.70	-14.06 to 0.45
Stat6i-Lip	**-12.58**	**3.70**	**-19.83 to -5.32**
**Time (weeks)**			
0	Reference	Reference	Reference
4	**-9.18**	**3.62**	**-16.27 to -2.09**
8	**-8.23**	**3.62**	**-15.32 to -1.14**
12	**-12.93**	**3.62**	**-20.02 to -5.84**
**Treatment x Time**			
Clod-Lip (4wk)	5.90	5.12	-4.13 to 15.93
Clod-Lip (8wk)	**10.83**	**5.12**	**0.80 to 20.85**
Clod-Lip (12wk)	**13.75**	**5.12**	**3.73 to 23.79**
Stat1i-Lip (4wk)	9.58	5.12	-0.45 to 19.61
Stat1i-Lip (8wk)	**14.12**	**5.12**	**4.09 to 24.14**
Stat1i-Lip (12wk)	2.77	5.12	-7.26 to 12.80
Stat6i-Lip (4wk)	**10.45**	**5.12**	**0.42 to 20.47**
Stat6i-Lip (8wk)	**14.43**	**5.12**	**4.40 to 24.46**
Stat6i-Lip (12wk)	**12.26**	**5.12**	**2.23 to 22.28**
**Intercept**	79.91	2.62	74.78 to 85.04
Random Effects			
**Variable**	**Variance**	**Standard Error**	**95% Confidence Intervals**
Intercept (all)	68.53	8.10	54.36 to 86.40
Intercept (by group)	3.10	5.10	-6.89 to 13.09
Exchangeable covariance matrix used to control for repeated measures			
